# Higher Dispositional Optimism Predicts Better Health-Related Quality of Life After Esophageal Cancer Surgery: A Nationwide Population-Based Longitudinal Study

**DOI:** 10.1245/s10434-021-10026-w

**Published:** 2021-04-19

**Authors:** Yangjun Liu, Erik Pettersson, Anna Schandl, Sheraz Markar, Asif Johar, Pernilla Lagergren

**Affiliations:** 1 Department of Molecular Medicine and Surgery, Karolinska Institutet, Karolinska University Hospital, Stockholm, Sweden; 2 Department of Medical Epidemiology and Biostatistics, Karolinska Institutet, Stockholm, Sweden; 3 Department of Anesthesiology and Intensive Care, Södersjukhuset, Stockholm, Sweden; 4 Department of Surgery and Cancer, Imperial College London, London, UK

## Abstract

**Purpose:**

To assess whether higher dispositional optimism could predict better health-related quality of life (HRQL) after esophageal cancer surgery.

**Methods:**

This Swedish nationwide longitudinal study included 192 patients who underwent esophagectomy for cancer. The exposure was dispositional optimism measured by the Life Orientation Test-Revised (LOT-R) at 1 year post-surgery. Patients were categorized into four subgroups (very low, moderately low, moderately high, and very high dispositional optimism) based on the quartile of the LOT-R sum score. The outcome was HRQL assessed by the European Organization for Research and Treatment of Cancer (EORTC) Quality of Life Questionnaire-Core 30 (QLQ-C30) and Quality of Life Questionnaire-Esophago-Gastric module 25 (QLQ-OG25) at 1, 1.5, and 2 years post-surgery. Linear mixed-effects models, adjusted for potential confounders, were used to examine the mean score difference (MSD) with 95% confidence interval of HRQL among the four patient subgroups.

**Results:**

Patients with very high dispositional optimism reported clinically relevantly better global quality of life, emotional function, and social function (MSD range 10–16) and less severe symptoms in pain, dyspnea, diarrhea, eating difficulty, anxiety, dry mouth, trouble with taste, worry about weight loss, and self-doubt about body image (MSD range − 9 to − 22) than patients with lower dispositional optimism. Patients with moderately high dispositional optimism reported clinically and statistically significantly better global quality of life (MSD 10) and less severe diarrhea (MSD − 9) than patients with lower dispositional optimism. Adjusted MSDs were constant over the three time points in all aspects except for eating difficulty.

**Conclusions:**

Measuring dispositional optimism could help identify patients at higher risk of poor HRQL recovery after esophageal cancer surgery.

**Supplementary Information:**

The online version contains supplementary material available at 10.1245/s10434-021-10026-w.

Esophageal cancer is a malignant tumor that ranks as the sixth leading cause of cancer death globally.^1^ Esophagectomy, often in combination with chemotherapy or chemoradiotherapy, is the main treatment approach with curative intent. Patients with esophagectomy for cancer usually suffer from substantially decreased health-related quality of life (HRQL), especially within 6 months post-surgery.^2^,^3^ From 1 year post-surgery, HRQL is reported to have recovered to a large extent.^2^,^3^ Previous studies have demonstrated that histology type, tumor stage, tumor location, operation approach, postoperative complications, and comorbidity are predictors of poor postoperative HRQL.^4^–^7^ However, patients with similar characteristics in these aspects still report varied HRQL, indicating that other factors, such as personality traits, might also have an influence.

Dispositional optimism is a relatively stable personality trait, which refers to the global expectation that more desirable than bad things will happen in the future.^8^ No previous study has assessed the association between dispositional optimism and HRQL among patients with esophageal cancer. Studies conducted among patients with other subtypes of cancer have shown that higher dispositional optimism is associated with better HRQL in several aspects, such as global quality of life, emotional function, social function, pain, and body image.^9^–^15^ However, its associations with other aspects including physical function, role function, and cognitive function were ambiguous, as some studies reported significant associations while others reported nonsignificant associations.^12^–^14^,^16^ In addition, one study found that the association between dispositional optimism and HRQL tends to attenuate when near death.^17^ Given that more than 50% of surgically treated patients with esophageal cancer die within 5 years post-surgery,^18^ it remains uncertain whether higher dispositional optimism could predict better HRQL in this population. Clarifying this predictive effect may help identify vulnerable patients who are at higher risk of suffering from poor HRQL after surgery, thus providing early and personalized interventions to patients in need. Moreover, as dispositional optimism can be increased via psychological interventions,^19^ if this predictive effect exists, it may also imply a potential intervention target to improve postoperative HRQL.

In this study, we aimed to use Swedish nationwide population-based longitudinal data to assess whether higher dispositional optimism predicts better HRQL after esophageal cancer surgery.

## Patients and Methods

### Study Design and Data Collection

Data for this longitudinal study were drawn from a prospective, ongoing Swedish nationwide cohort study entitled Oesophageal Surgery on Cancer patients-Adaptation and Recovery (OSCAR). Detailed description of the OSCAR study has been presented elsewhere.^20^ In brief, it includes 1-year esophageal cancer survivors without cognitive dysfunction who underwent curative-intent esophagectomy in Sweden from 1 January 2013 onwards (response rate around 66%).^20^ Eligible patients are identified through collaboration with pathology departments at eight hospitals performing esophagectomy in Sweden.^20^ Survival information is collected through linkage to the Swedish Register of the Total Population and the Swedish cause of death register.^20^ Follow-ups on patient-reported outcomes start from 1 year and last until 5 years post-surgery through personal interview or mailing paper questionnaires.^20^ In addition, patients’ demographics are retrieved from the Swedish national health data registries and the Swedish Longitudinal Integration Database for Health Insurance and Labor Market Studies.^20^ Clinical data are obtained from medical charts, the Swedish Patient Registry, and the Swedish Cancer Registry.^20^ The study was approved by the Regional Ethical Review Board in Stockholm, Sweden (diary number 2013/844-31/1), and written consent was obtained from all participants before inclusion.

The present study included patients who underwent esophagectomy for cancer between 1 January 2013 and 28 February 2018, and incorporated three follow-up time points at 1, 1.5, and 2 years post-surgery. Patients who died during the follow-up period, had psychiatric history, or were diagnosed with noncancerous neoplasm (dysplasia) were excluded.

### Exposure: Dispositional Optimism

Dispositional optimism was measured at 1 year post-surgery using the Swedish version of Life Orientation Test-Revised (LOT-R).^21^,^22^ LOT-R comprises three positively worded items and three negatively worded items,^21^,^22^ and asks patients to report their agreement with each item on a five-point Likert scale ranging from 0 (“strongly disagree”) to 4 (“strongly agree”).^22^

Due to the ambiguous dimensionality of LOT-R and absence of psychometric study in patients with esophageal cancer, we conducted a series of confirmatory factor analyses to assess the factor structure of LOT-R. Detailed results can be found elsewhere.^23^ Because the first negatively worded item had negative loading in the best-fit model and its response distribution was bimodal, we removed this item and adopted the model assuming one factor (dispositional optimism) with correlated errors between the two reversed negatively worded items. The internal reliability estimated by McDonald’s omega for this model was 0.49 [95% bootstrapped confidence interval (CI) 0.31–0.62].

The remaining five items of LOT-R were summed, of which the two negatively worded items were reversed. A higher sum score represents higher dispositional optimism. Based on the quartile of the sum score, patients were categorized into four subgroups with very low, moderately low, moderately high, and very high dispositional optimism.

### Outcome: HRQL

HRQL was measured at 1, 1.5, and 2 years post-surgery using the European Organization for Research and Treatment of Cancer Quality of Life Questionnaire-Core 30 (EORTC QLQ-C30) and disease site-specific (Esophago-Gastric) module EORTC QLQ-OG25.^24^,^25^ Both questionnaires are validated in Swedish and have demonstrated good psychometric properties.^24^,^25^

The EORTC QLQ-C30 contains 30 items with Likert scaling and measures HRQL aspects of cancer patients in general. It consists of one global quality-of-life subscale, five functional subscales (physical, role, emotional, social, and cognitive), three symptom subscales (fatigue, pain, and nausea/vomiting), and six single items (dyspnea, appetite loss, insomnia, constipation, diarrhea, and financial difficulty).^24^ Items in the global quality-of-life subscale range from 1 (“very poor”) to 7 (“excellent”), while items in other subscales score from 1 (“not at all”) to 4 (“very much”).^24^

The EORTC QLQ-OG25 is a 25-item esophagogastric cancer-specific questionnaire, which comprises six symptom subscales (dysphagia, eating difficulty, reflux, odynophagia, pain and discomfort, and anxiety) and ten single items (eating in front of others, dry mouth, trouble with taste, trouble swallowing saliva, choked when swallowing, trouble with coughing, trouble talking, weight loss, body image, and hair loss).^25^ All items are scored on a four-point Likert scale ranging from 1 (“not at all”) to 4 (“very much”).^25^

Missing values in both questionnaires were handled according to the EORTC Scoring Manual,^26^ and the raw score of each HRQL subscale was transformed to a linear scale of 0–100.^26^ A higher score represents better function/global quality of life or higher symptom burden.^26^ In addition, a single summary score for the EORTC QLQ-C30 was calculated according to the EORTC guideline.^27^

### Statistical Analysis

Sociodemographic and clinical characteristics of all included patients as well as the four patient subgroups were summarized. We compared the overall mean of the LOT-R sum score between patients with different sociodemographic and clinical characteristics using *t*-test or analysis of variance (ANOVA). Linear mixed-effects model was used to examine the mean score differences (MSDs) of HRQL among the four patient subgroups with hierarchical dispositional optimism levels. Covariates include time (1, 1.5, and 2 years post-surgery), age (continuous variable), sex (female or male), cohabitation status (non-cohabitating or cohabitating), education level (9-year compulsory school, upper secondary school, or higher education), Charlson Comorbidity Index^28^ (0 or ≥ 1), tumor stage (complete regress after neoadjuvant therapy/I, II, or III–IV), histology (squamous cell carcinoma or adenocarcinoma), postoperative Clavien–Dindo complication score (none, I–II, or III–IV), and weight change after surgery (continuous variable). Sociodemographic factors were included because they are potential confounders associated with both dispositional optimism and HRQL.^29^,^30^ Although no previous studies have suggested that clinical factors affect dispositional optimism, we included these factors as covariates to increase the estimation precision because they are strongly associated with postoperative HRQL.^4^–^7^,^31^ In addition, the interaction effect between dispositional optimism and time was examined using the Wald test. In all models, four covariance matrices (unstructured, independent, exchangeable, and identity) were compared using the Bayesian information criterion (BIC), and the one with lower BIC is preferred because it indicates better balance between goodness of fit and parsimony.^32^

HRQL measures several aspects; to minimize the type I error due to multiple comparisons, we tested the statistical significance of adjusted MSD only if it had clinical relevance based on evidence-based guidelines.^33^–^36^ The adjusted MSD of HRQL aspects measured by EORTC QLQ-C30 has four grades: *trivial* (circumstances unlikely to have any clinical relevance or where there was no difference), *small* (subtle but clinically relevant), *medium* (likely to be clinically relevant but to a lesser extent), or *large* (of unequivocal clinical relevance).^33^,^34^ We regarded medium and large differences as clinically relevant in the present study. For other HRQL aspects, an adjusted MSD of ≥ 10 was considered clinically relevant.^35^,^36^

Stata 13 and SAS 9.4 were used for the statistical analyses. All 95% confidence intervals (CIs) were two sided.

## Results

### Study Participants

**Fig. 1:**
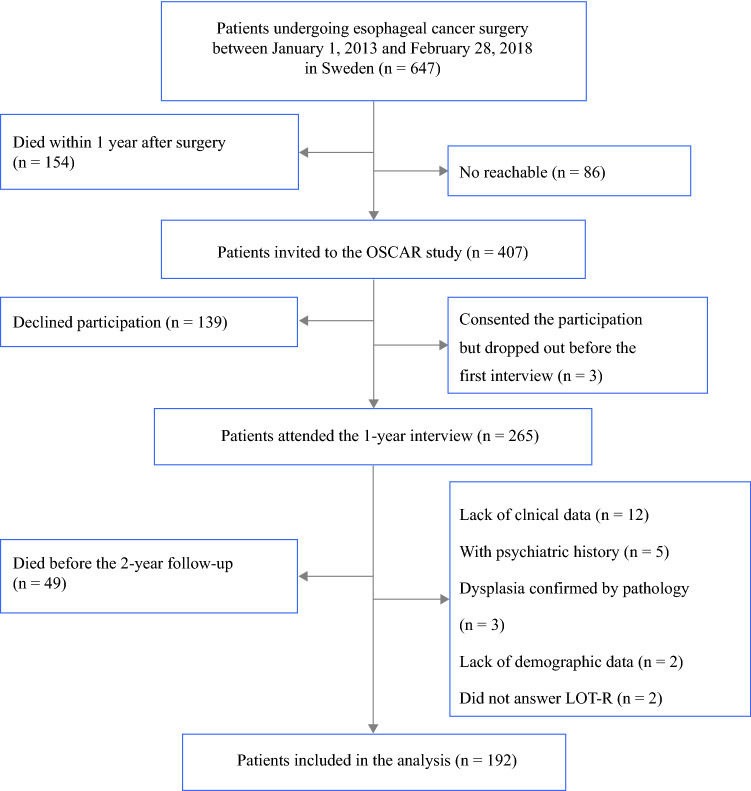
Flowchart of patient selection for inclusion. *OSCAR study* Oesophageal Surgery on Cancer patients-Adaptation and Recovery (OSCAR), a prospective, ongoing Swedish-nationwide cohort study, *LOT-R* Life Orientation Test-Revised

Figure 1 displays the detailed process of patient selection. Between 1 January 2013 and 28 February 2018, 647 patients underwent curative-intent esophageal cancer surgery in Sweden. Of these, 407 patients were invited to participate in the study, and 265 (65%) patients consented to participation and finished the first (1-year) interview. Nonparticipation was mainly due to unwillingness, poor health, and cancer recurrence.^20^ In addition, 73 patients were further excluded due to death during follow-up (*n* = 49), psychiatric history before surgery (*n* = 5), noncancerous histology (*n* = 3), and lack of essential data (*n* = 16), leaving 192 patients included in the analysis. Among them, 170 and 156 patients answered the 1.5- and 2-year questionnaire, respectively.

### Characteristics of Participants

Table 1 presents the sociodemographic and clinical characteristics of the 192 patients. Mean age at surgery was 66.3 years [standard deviation (SD) 8.5 years, range 38.2–83.7 years). Most patients were male (85.4%) and married/cohabitating (77.1%). The mean of the LOT-R sum score was 15.2 (SD 3.0) with range of 6–20. Based on the quartile of LOT-R sum score, patients were categorized into four subgroups with hierarchical dispositional optimism levels: very low (*n* = 48, LOT-R sum score range 6–13), moderately low (*n* = 51, LOT-R sum score range 14–15), moderately high (*n* = 45, LOT-R sum score range 16–17), and very high (*n* = 48, LOT-R sum score range 19–20). Sociodemographic and clinical characteristics of the four patient subgroups are presented in Table 1. Patients with different sociodemographic and clinical characteristics reported similar LOT-sum scores (Supplementary Table S1).

**Table 1:** Characteristics of all included patients with esophagectomy for cancer and four patient subgroups with hierarchical dispositional optimism levels

	All included patients (*n* = 192)	Dispositional optimism level			
		Very low (*n* = 48)	Moderately low (*n* = 51)	Moderately high (*n* = 45)	Very high (*n* = 48)
*LOT-R sum score*					
Mean ± SD	15.2 ± 3.0	11.3 ± 1.6	14.5 ± 0.5	16.5 ± 0.5	18.9 ± 0.9
Range	[6, 20]	[6, 13]	[14, 15]	[16, 17]	[18, 20]
*Age (years)*					
Mean ± SD	66.3 ± 8.5	64.2 ± 9.9	67.9 ± 8.3	65.7 ± 7.9	67.2 ± 7.3
Range	[38.2, 83.7]	[38.2, 82.0]	[41.5, 83.7]	[49.9, 80.5]	[50.0, 80.0]
*Sex*					
Female	28 (14.6)	4 (8.3)	7 (13.7)	7 (15.6)	10 (20.8)
Male	164 (85.4)	44 (91.7)	44 (86.3)	38 (84.4)	38 (79.2)
*Cohabitation status*					
Non-cohabitating	44 (22.9)	13 (27.1)	13 (25.5)	10 (22.2)	8 (16.7)
Cohabitating	148 (77.1)	35 (72.9)	38 (74.5)	35 (77.8)	40 (83.3)
*Education level*					
Nine-year compulsory school	48 (25.0)	11 (22.9)	16 (31.4)	11 (24.4)	10 (20.8)
Upper secondary school	85 (44.3)	24 (50.0)	19 (37.3)	22 (48.9)	20 (41.7)
Higher education	59 (30.7)	13 (27.1)	16 (31.4)	12 (26.7)	18 (37.5)
*Neoadjuvant therapy*					
Yes	158 (82.3)	41 (85.4)	38 (74.5)	37 (82.2)	42 (87.5)
No	34 (17.7)	7 (14.6)	13 (25.5)	8 (17.8)	6 (12.5)
*Operation approach*					
Total minimally invasive esophagectomy	52 (27.1)	18 (37.5)	11 (21.6)	7 (15.6)	16 (33.3)
Hybrid minimally invasive esophagectomy	63 (32.8)	18 (37.5)	12 (23.5)	17 (37.8)	16 (33.3)
Open esophagectomy	77 (40.1)	12 (25.0)	28 (54.9)	21 (46.7)	16 (33.3)
*Tumor stage*					
Complete regression after neoadjuvant therapy or I	71 (37.0)	15 (31.3)	27 (52.9)	12 (26.7)	17 (35.4)
II	62 (32.3)	20 (41.7)	11 (21.6)	16 (35.6)	15 (31.3)
III–IV	59 (30.7)	13 (27.1)	13 (25.5)	17 (37.8)	16 (33.3)
*Tumor histology*					
Adenocarcinoma	163 (84.9)	43 (89.6)	39 (76.5)	43 (95.6)	38 (79.2)
Squamous cell carcinoma	29 (15.1)	5 (10.4)	12 (23.5)	2 (4.4)	10 (20.8)
*Postoperative complications (Clavien–Dindo grade)*					
No complications	69 (35.9)	21 (43.8)	15 (29.4)	17 (37.8)	16 (33.3)
I–II	54 (28.1)	12 (25.0)	18 (35.3)	14 (31.1)	10 (20.8)
III–IV	69 (35.9)	15 (31.3)	18 (35.3)	14 (31.1)	22 (45.8)
*Charlson comorbidity index*					
0	94 (49.0)	18 (37.5)	25 (49.0)	27 (60.0)	24 (50.0)
1	60 (31.3)	19 (39.6)	16 (31.4)	11 (24.4)	14 (29.2)
≥ 2	38 (19.8)	11 (22.9)	10 (19.6)	7 (15.6)	10 (20.8)

All values are *n* (%) unless otherwise stated, and the percentage is rounded up, which in some cases gives a sum not equaling to 100%

*LOT-R* life orientation test-revised

### Time-Invariant Predictive Effect of Dispositional Optimism on HRQL

**Fig. 2:**
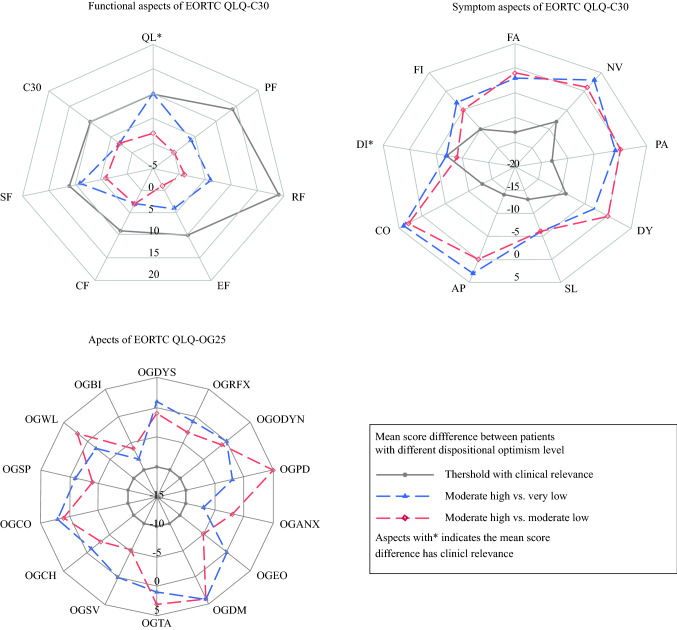
Mean score differences in health-related quality of life aspects between patients with moderately high dispositional optimism and lower (very low/moderately low) dispositional optimism over the three assessment time points (1, 1.5, and 2 years after esophageal cancer surgery). *EORTC QLQ-C30* European Organization for Research and Treatment of Cancer Quality of Life Questionnaire-Core 30, *QL* global quality of life, *PF* physical function, *RF* role function, *EF* emotional function, *CF* cognitive function, *SF* social function, *C30* EORTC QLQ-C30 summary score, *FA* fatigue, *NV* nausea/vomiting, *PA* pain, *DY* dyspnea, *SL* insomnia, *AP* appetite loss, *CO* constipation, *DI* diarrhea, *FI* financial difficulty, *EORTC QLQ-OG25* European Organization for Research and Treatment of Cancer Quality of Life Questionnaire-Esophago-Gastric module 25, *OGDYS* dysphagia, *OGRFX* reflux, *OGODYN* odynophagia, *OGPD* pain and discomfort, *OGANX* anxiety, *OGEO* eating with others, *OGDM* dry mouth, *OGTA* trouble with taste, *OGSV* trouble with swallowing saliva, *OGCH* choked when swallowing, *OGCO* trouble with coughing, *OGSP* trouble talking, *OGWL* worry about weight loss, *OGBI* self-doubt regarding body image

**Fig. 3.**
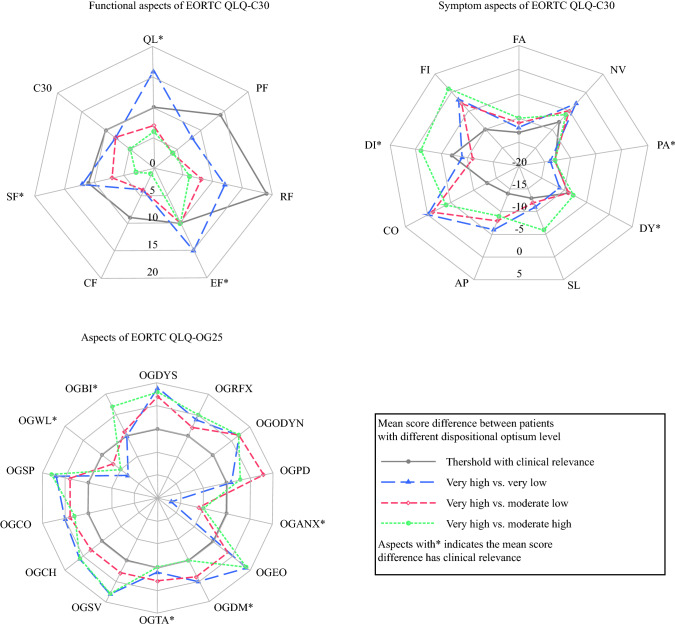
Mean score differences in health-related quality of life aspects between patients with very high dispositional optimism and lower (very low/moderately low/moderately high) dispositional optimism over the three assessment time points (1, 1.5, and 2 years after esophageal cancer surgery). *EORTC QLQ-C30* European Organization for Research and Treatment of Cancer Quality of Life Questionnaire-Core 30, *QL* global quality of life, *PF* physical function, *RF* role function, *EF* emotional function, *CF* cognitive function, *SF* social function, *C30* EORTC QLQ-C30 summary score, *FA* fatigue, *NV* nausea/vomiting, *PA* pain, *DY* dyspnea, *SL* insomnia, *AP* appetite loss, *CO* constipation, *DI* diarrhea, *FI* financial difficulty, *EORTC QLQ-OG25* European Organization for Research and Treatment of Cancer Quality of Life Questionnaire-Esophago-Gastric module 25, *OGDYS* dysphagia, *OGRFX* reflux, *OGODYN* odynophagia, *OGPD* pain and discomfort, *OGANX* anxiety, *OGEO* eating with others, *OGDM* dry mouth, *OGTA* trouble with taste, *OGSV* trouble with swallowing saliva, *OGCH* choked when swallowing, *OGCO* trouble with coughing, *OGSP* trouble talking, *OGWL* worry about weight loss, *OGBI* self-doubt regarding body image

In almost all HRQL aspects except for eating difficulty, the predictive effect of dispositional optimism on HRQL was not modified by time. Over the three assessment time points, there was no clinically relevant difference between patients with very low and moderately low dispositional optimism in any HRQL aspect (Supplementary Table S2). However, compared with patients with very low and moderately low dispositional optimism, patients with moderately high dispositional optimism reported clinically relevantly and statistically significantly better global quality of life (MSD 10, 95% CI 4–17) and less severe diarrhea (MSD –9, 95% CI − 18 to − 1; Fig. 2; Supplementary Table S2). Compared with patients with very low, moderately low, and moderately high dispositional optimism, patients with very high dispositional optimism reported clinically relevantly better global quality of life, emotional function, and social function (MSD range 10–16; Fig. 3; Supplementary Table S2) and less severe symptoms of pain, dyspnea, diarrhea, anxiety, dry mouth, trouble with taste, worry about weight loss, and self-doubt about body image (MSD range − 9 to − 22; Fig. 3; Supplementary Table S2). However, the MSDs for the aspects of dry mouth and trouble with taste were marginal (MSD − 10) and not statistically significant (95% CI − 21 to 1 and − 20 to + 0, respectively; Supplementary Table S2).

### Time-Varying Predictive Effect of Dispositional Optimism on HRQL

On the eating difficulty subscale, the predictive effect of dispositional optimism varied over time (interaction effect, *p* = 0.012). There was no clinically relevant difference between patients with moderately low and very low dispositional optimism, or between patients with moderately high and lower (very low/moderately low) dispositional optimism (Table 2). However, patients with very high dispositional optimism reported clinically relevant and statistically significant less eating difficulty than patients with lower (very low, moderately low, and moderately high) dispositional optimism, even though the MSDs varied at different time points (Table 2).

**Table 2.** Mean score difference with 95% confidence interval (CI) in eating difficulty at 1, 1.5, and 2 years after esophageal cancer surgery between patients with different dispositional optimism levels

	Time (years)	Mean score difference (95% CI)					
		Moderately low	Moderately high		Very high		
		versus very low	versus very low	versus moderately low	versus very low	versus moderately low	versus moderately high
Eating difficulty	1	8 (+0, 17)	4 (− 5, 12)	− 5 (− 13, 4)	− 4 (− 13, 4)	− **13 (**− **21,** − **4)**	− 8 (− 16, 1)
	1.5	7 (− 2, 16)	7 (− 2, 16)	0 (− 8, 9)	− 3 (− 12, 6)	− **10 (**− **19,** − **1)**	− **10 (**− **19,** − **2)**
	2	0 (− 10, 10)	− 7 (− 18, 3)	− 7 (− 17, 3)	− **11 (**− **21,** − **1)**	− **11 (**− **21,** − **1)**	− 4 (− 14, 7)

Values marked in bold have both clinical and statistical significance

Mean score difference rounded up to the nearest integer

## Discussion

This study showed that, compared with patients with lower dispositional optimism, patients with higher dispositional optimism reported better HRQL at 1, 1.5, and 2 years after esophageal cancer surgery, in the aspects of global quality of life, emotional function, social function, anxiety, pain, and body image, which is in line with previous studies conducted among patients with other subtypes of cancer.^9^–^15^ However, this study further found that dispositional optimism predicted fewer self-reported problems in dyspnea, diarrhea, dry mouth, trouble with taste, and worry about weight loss after esophageal cancer surgery.

The potential mechanisms for these observed associations might be related to coping, goal adjustment, and social support.^37^–^40^ Previous studies have shown that patients with higher dispositional optimism tend to adopt more effective coping and goal adjustment strategies.^37^–^39^ When the challenge is controllable, people with higher dispositional optimism tend to make every possible effort to overcome it, but if the challenge is uncontrollable, they also tend to disengage from the unattainable goals and adapt to unfavorable situations more quickly via using emotional acceptance and positive reinterpretation.^37^,^38^ Therefore, patients with higher dispositional optimism may be more persistent in pursing beneficial lifestyle,^41^ such as obeying the special postoperative dietary instructions, quitting smoking, and undertaking more physical exercise,^41^ which might help them reduce the symptoms of diarrhea and eating difficulty,^42^ and relieve their worry about weight loss. In addition, after esophageal cancer diagnosis and surgery, some life goals may become unattainable for patients. Timely disengagements from unrealistic goals and reengagement in new achievable goals can help patients avoid accumulating negative experience and reduce rumination,^39^ which might lead to better emotional function as well as less anxiety and pain.^38^,^39^ Given that psychological distress is a potential cause of dyspnea,^43^ better emotional function might further help optimistic patients reduce dyspnea symptom. Additionally, more optimistic people are more likely to have higher perceptions of available social support as well as actually receive higher supportiveness from significant others,^40^ thus leading to better social function and less self-doubt about body image. The combined beneficial effects of high dispositional optimism on the above HRQL aspects might further contribute to better global quality of life.

The observed predictive effect of dispositional optimism on HRQL among patients with esophagectomy for cancer has both clinical and research implications. It may help identify patients at higher risk of suffering from persistently impaired HRQL after esophageal cancer surgery, thus providing tailored follow-up and timely interventions to improve their postoperative HRQL. Moreover, although dispositional optimism is relatively stable, it can be increased via psychological interventions such as cognitive behavior therapy and the Best Possible Self exercise.^19^ The findings of this study suggest that increasing dispositional optimism might be a potential intervention target to improve postoperative HRQL. In addition, given that poor HRQL recovery is associated with higher mortality,^44^ the predictive value of dispositional optimism on HRQL may also imply its potential predictive effect on survival, and future studies on this topic are warranted.

To the best of the authors’ knowledge, this is the first study examining the predictive effect of dispositional optimism on HRQL after esophageal cancer surgery. The study reduced confounding bias through adjusting for several potential confounders. HRQL was measured comprehensively using both a cancer general questionnaire (EORTC QLQ-C30) and a disease-specific module (EORTC QLQ-OG25). Moreover, the clinical relevance of adjusted MSD was evaluated according to the evidence-based guidelines, and its statistical significance was tested only if it had clinical significance, which not only decreased the risk of chance findings but also ensured the clinical relevance of the results. Additionally, the nationwide population-based longitudinal study design facilitated the generalizability of the findings.

This study also has some limitations. First, because the OSCAR study focuses on patients who have survived for at least 1 year after esophagectomy, we measured dispositional optimism at 1 year post-surgery and not at time of cancer diagnosis or surgery. The observed predictive effect of dispositional optimism on HRQL should be interpreted in light of the assessment time point, even though dispositional optimism remains relatively stable over time and across stressful situations including receiving cancer diagnosis and surgery,^45^,^46^ and surgical factors were not associated with dispositional optimism based on our data. Second, we categorized patients into four subgroups according to the quartile of LOT-R sum score. Misclassification might happen due to the measurement error in the LOT-R, which can cause potential bias in either direction.^47^ Third, patients with lower dispositional optimism and poor HRQL might be more likely to decline to participate in the study and not answer the follow-up questionnaires, which could make the observed associations underestimated. Last but not least, prediction does not equal causation. The observed association might be due to the effect of unmeasured confounders such as genetic factors,^48^,^49^ and whether increasing dispositional optimism could improve HRQL needs to be examined by future interventional studies.

In conclusion, this study showed that higher dispositional optimism predicted better HRQL at 1, 1.5, and 2 years post-surgery in several aspects among patients with esophagectomy for cancer. The predictive value of dispositional optimism may help identify high-risk patients with poor HRQL recovery after esophageal cancer surgery, leading to timely and tailored interventions to patients in need, and therefore contribute to the improvement of postoperative HRQL and probably even survival.

## Supplementary Information

Below is the link to the electronic supplementary material.

Supplementary file1 (DOCX 32 KB)
