# Supplementary material for: Higher Dispositional Optimism Predicts Better Health-Related Quality of Life After Esophageal Cancer Surgery: A Nationwide Population-Based Longitudinal Study
Source: Ann Surg Oncol. 2021 Apr 19;28(12):7196–205. doi: 10.1245/s10434-021-10026-w (PMC8521517; doi:10.1245/s10434-021-10026-w)
Supplement: Supplementary file 1 — Supplementary file1 (DOCX 32 KB) [file 10434_2021_10026_MOESM1_ESM.docx]

**Table S1.** LOT-R sum score in 1-year esophageal cancer survivors with different sociodemographic and clinical characteristics

|  | Number (%)  (n = 192) | LOT-R sum score | | |
| --- | --- | --- | --- | --- |
|  |  | Mean | Standard deviation | P value |
| **Age** |  |  |  |  |
| < 60 | 44 (22.9) | 15.1 | 3.3 | 0.95 |
| 60-74 | 119 (62.0) | 15.3 | 2.9 |  |
| ≥75 | 29 (15.1) | 15.2 | 2.6 |  |
| **Sex** |  |  |  |  |
| Female | 28 (14.6) | 15.9 | 3.0 | 0.19 |
| Male | 164 (85.4) | 15.1 | 3.0 |  |
| **Cohabitation status** |  |  |  |  |
| Non-cohabitating | 44 (22.9) | 14.6 | 2.7 | 0.13 |
| Cohabitating | 148 (77.1) | 15.4 | 3.0 |  |
| **Education level** |  |  |  |  |
| Nine-year compulsory school | 48 (25.0) | 15.2 | 2.8 | 0.91 |
| Upper secondary school | 85 (44.3) | 15.2 | 3.1 |  |
| Higher education | 59 (30.7) | 15.4 | 3.0 |  |
| **Neoadjuvant therapy** | | | | |
| Yes | 158 (82.3) | 15.2 | 3.1 | 0.94 |
| No | 34 (17.7) | 15.2 | 2.5 |  |
| **Operation approach** | | | | |
| Total minimally invasive esophagectomy | 52 (27.1) | 15.0 | 3.2 | 0.79 |
| Hybrid minimally invasive esophagectomy | 63 (32.8) | 15.2 | 3.2 |  |
| Open esophagectomy | 77 (40.1) | 15.4 | 2.5 |  |
| **Tumor stage** | | | | |
| Complete regression after neoadjuvant therapy/ I | 71 (37.0) | 15.1 | 2.7 | 0.66 |
| II | 62 (32.3) | 15.1 | 3.2 |  |
| III- IV | 59 (30.7) | 15.5 | 3.0 |  |
| **Tumor histology** | | | | |
| Adenocarcinoma | 163 (84.9) | 15.2 | 2.9 | 0.74 |
| Squamous cell carcinoma | 29 (15.1) | 15.4 | 3.2 |  |
| **Postoperation complications (Clavien–Dindo grade)** | | | | |
| No complication | 69 (35.9) | 15.1 | 3.0 | 0.43 |
| I–II | 54 (28.1) | 14.9 | 2.9 |  |
| III–IV | 69 (35.9) | 15.6 | 2.9 |  |
| **Charlson comorbidity index** | | | | |
| 0 | 94 (49.0) | 15.4 | 3.2 | 0.71 |
| 1 | 60 (31.3) | 15.0 | 2.7 |  |
| ≥2 | 38 (19.8) | 15.1 | 2.7 |  |

Note. LOT-R: life orientation test-revised.

**Table S2.** Mean score of health related quality of life (HRQL) for patients with “very low dispositional optimism” at 1 year post-surgery and mean score difference with a 95% confidence interval (CI) in HRQL over the three assessment time points (1, 1.5 and 2 years post-surgery) between postoperative esophageal cancer patients with different dispositional optimism levels

|  | **Mean score**  **(95% CI)** |  | **Mean score difference (95% CI)** | | | | | | | |
| --- | --- | --- | --- | --- | --- | --- | --- | --- | --- | --- |
|  | **Very low** |  | **Moderately low** |  | **Moderately high** | |  | **Very high** | | |
|  | 1 year |  | vs. very low |  | vs. very low | vs. moderately low |  | vs. very low | vs. moderately low | vs. moderately high |
| **EORTC QLQ-C30** |  |  |  |  |  |  |  |  |  |  |
| Global quality of life | 61 (56, 66) |  | 9 (2, 15) |  | **10 (4, 17)** | 2 (-5, 8) |  | **16 (9, 23)** | 7 (1, 14) | 6 (-1, 12) |
| Physical function | 82 (78, 86) |  | 4 (-2, 10) |  | 4 (-1, 10) | 0 (-5, 6) |  | 8 (2, 14) | 4 (-1, 10) | 4 (-2, 10) |
| Role function | 76 (70, 82) |  | 5 (-3, 13) |  | 6 (-2, 14) | 1 (-6, 9) |  | 12 (5, 20) | 8 (+0, 15) | 6 (-2, 14) |
| Emotional function | 78 (74, 83) |  | 5 (-1, 12) |  | 4 (-2, 11) | -1 (-8, 5) |  | **15 (9, 21)** | **10 (3, 16)** | **10 (4, 17)** |
| Cognitive function | 84 (79, 89) |  | 0 (-7, 7) |  | 3 (-4, 10) | 3 (-4, 10) |  | 4 (-3, 11) | 4 (-3, 11) | 1 (-6, 8) |
| Social function | 76 (70, 82) |  | 5 (-3, 14) |  | 9 (+0, 17) | 4 (-5, 12) |  | **12 (4, 21)** | 7 (-1, 15) | 3 (-5, 12) |
| Fatigue | 33 (27, 39) |  | -1 (-9, 7) |  | -2 (-10, 6) | -1 (-9, 7) |  | -12 (-20, -4) | -11 (-18, -3) | -10 (-18, -2) |
| Nausea/Vomiting | 13 (8, 18) |  | 1 (-5, 8) |  | 3 (-4, 9) | 1 (-6, 8) |  | -3 (-10, 4) | -5 (-11, 2) | -6 (-13, 1) |
| Pain | 22 (16, 28) |  | -1 (-9, 8) |  | -1 (-9, 7) | 0 (-8, 8) |  | **-14 (-22, -5)** | **-13 (-21, -5)** | **-13 (-21, -5)** |
| Dyspnea | 32 (25, 38) |  | -2 (-11, 7) |  | -3 (-12, 7) | 0 (-10, 9) |  | **-11 (-20, -2)** | ***-9 (-18, +0)*** | -8 (-18, 1) |
| Insomnia | 26 (19, 34) |  | 1 (-10, 11) |  | -6 (-16, 5) | -6 (-16, 4) |  | -11 (-22, -1) | -12 (-22, -2) | -6 (-16, 5) |
| Appetite loss | 17 (10, 24) |  | 2 (-8, 12) |  | 3 (-7, 12) | 0 (-9, 10) |  | -6 (-16, 4) | -8 (-18, 1) | -9 (-19, 1) |
| Constipation | 6 (+0, 11) |  | 1 (-6, 8) |  | 4 (-3, 11) | 3 (-4, 10) |  | 0 (-7, 7) | -1 (-8, 5) | -4 (-11, 2) |
| Diarrhea | 23 (17, 30) |  | 2 (-7, 11) |  | -7 (-16, 1) | **-9 (-18, -1)** |  | **-9 (-18, -0)** | **-11 (-19, -2)** | -1 (-10, 7) |
| Financial difficulties | 10 (4, 16) |  | 2 (-6, 9) |  | -3 (-11, 5) | -5 (-13, 3) |  | -2 (-10, 6) | -3 (-11, 4) | 1 (-7, 9) |
| Summary score | 79 (76, 83) |  | 0 (-5, 5) |  | 3 (-2, 8) | 3 (-2, 7) |  | 8 (4, 13) | 8 (4, 13) | 5 (1, 10) |
| **EORTC QLQ-OG25** |  |  |  |  |  |  |  |  |  |  |
| Dysphagia | 7 (3, 11) |  | 2 (-4, 7) |  | 1 (-4, 7) | -1 (-6, 5) |  | -1 (-6, 5) | -3 (-8, 3) | -2 (-8, 3) |
| Reflux | 26 (19, 33) |  | 2 (-8, 11) |  | -1 (-10, 9) | -3 (-12, 7) |  | -6 (-16, 4) | -8 (-17, 1) | -5 (-15, 4) |
| Odynophagia | 13 (8, 18) |  | 0 (-6, 7) |  | 0 (-7, 6) | -1 (-7, 6) |  | -3 (-10, 3) | -3 (-10, 3) | -3 (-9, 4) |
| Pain and discomfort | 29 (22, 35) |  | -7 (-16, 2) |  | -2 (-11, 7) | 5 (-4, 14) |  | -9 (-18, +0) | -2 (-10, 7) | -7 (-16, 2) |
| Anxiety | 42 (36, 49) |  | -5 (-15, 4) |  | -7 (-16, 2) | -2 (-11, 7) |  | **-22 (-31, -12)** | **-16 (-25, -7)** | **-15 (-24, -5)** |
| Eating with others | 7 (1, 13) |  | 5 (-3, 13) |  | 0 (-9, 8) | -5 (-13, 3) |  | -1 (-9, 7) | -6 (-14, 2) | -1 (-9, 7) |
| Dry mouth | 28 (20, 36) |  | 0 (-11, 12) |  | 4 (-7, 16) | 4 (-7, 15) |  | -5 (-17, 6) | -6 (-17, 5) | ***-10 (-21, 1)*** |
| Trouble with taste | 17 (10, 24) |  | -2 (-11, 8) |  | 1 (-9, 11) | 3 (-7, 13) |  | -9 (-19, 1) | -7 (-17, 2) | ***-10 (-20, +0)*** |
| Trouble swallowing saliva | 6 (1, 10) |  | 5 (-2, 11) |  | 0 (-7, 6) | -5 (-11, 1) |  | -2 (-9, 4) | -7 (-13, -1) | -2 (-9, 4) |
| Choked when swallowing | 14 (9, 20) |  | 3 (-5, 11) |  | -1 (-9, 8) | -3 (-11, 5) |  | -4 (-12, 4) | -7 (-15, 1) | -4 (-12, 4) |
| Trouble with coughing | 35 (27, 43) |  | 1 (-9, 12) |  | 2 (-9, 13) | 1 (-10, 12) |  | -5 (-16, 6) | -6 (-17, 5) | -7 (-18, 4) |
| Trouble talking | 7 (2, 12) |  | 2 (-4, 9) |  | -1 (-8, 6) | -4 (-10, 3) |  | -3 (-10, 4) | -6 (-12, 1) | -2 (-9, 5) |
| Weight loss | 27 (19, 34) |  | -3 (-14, 7) |  | -2 (-12, 9) | 2 (-9, 12) |  | **-17 (-27, -6)** | **-13 (-23, -3)** | **-15 (-26, -5)** |
| Body image | 17 (10, 23) |  | -1 (-10, 8) |  | -8 (-17, 1) | -6 (-15, 3) |  | **-10 (-19, -1)** | -9 (-18, -0) | -3 (-12, 6) |

Note. Values marked in bold have both clinical and statistical significance; values marked in bold and italic have only clinical relevance but no statistical significance. Mean score and mean score difference rounded up to the nearest integer. EORTC QLQ-C30, European Organization for Research and Treatment of Cancer Quality of Life Questionnaire - Core 30; EORTC QLQ-OG25, European Organization for Research and Treatment of Cancer Quality of Life Questionnaire - Esophago-Gastric module 25.
